# Changing smoking habits and the occurrence of lung cancer in Sweden—a population analysis

**DOI:** 10.1093/eurpub/ckae050

**Published:** 2024-03-22

**Authors:** Bengt Järvholm, Linnea Hedman, Maréne Landström, Per Liv, Alex Burdorf, Kjell Torén

**Affiliations:** Department of Public Health and Clinical Medicine, Umeå University, Umeå, Sweden; Department of Public Health and Clinical Medicine, Umeå University, Umeå, Sweden; Pathology Section, Department of Medical Biosciences, Umeå University, Umeå, Sweden; Department of Public Health and Clinical Medicine, Umeå University, Umeå, Sweden; Department of Public Health, Erasmus MC, Rotterdam, The Netherlands; Section of Occupational and Environmental Medicine, University of Gothenburg, Gothenburg, Sweden

## Abstract

**Background:**

The objective is to estimate the importance of the decrease of smoking habits in Sweden for the occurrence of lung cancer.

**Methods:**

The change in smoking habits in the general population was retrieved from surveys and on taxation of sale of cigarettes. We used data from the Swedish Cancer Register on incidence of lung cancer between 1970 and 2021, stratified for sex, age and cell type, and compared the occurrence overtime in ages between 40 and 84 years.

**Results:**

The sale of cigarettes peaked in 1980 to 1800 cigarettes per person and decreased to 600 per person in 2021. The change in incidence rates of squamous cell cancer and other cell types varied over time, sex, and age in a pattern that partly seems to be explained by change in the prevalence of daily smokers. The incidence of adenocarcinoma was similar in men and women 1970–2021 and increased, e.g. for women and men 75–79 years of age from around 20 cases in early 1970s to around 120 cases per 100 000 person-years in the 2020s.

**Conclusions:**

Our data indicate that the risk of lung cancer several years after smoking cessation is less favourable than previously studies have indicated. There is a similar increase in the incidence of adenocarcinoma in men and women which is hard to explain only with changing smoking habits. The change from non-filter to filter cigarettes in the 1960s–1970s may be a contributing factor.

## Introduction

Tobacco smoking was established as an important cause of lung cancer in the 1950s.^1^ Studies of smokers showed that the risk of lung cancer increased by amount smoked and decreased after smoking cessation.^2^ It was estimated that a male smoker that stopped smoking at 50 years of age decreased the cumulative risk of lung cancer at age 75 years from 15.9% to 3%, i.e. a reduction of about 80%.^3^ Reviews from the early 1980s showed that decreasing tobacco smoking was the most important measure to prevent the occurrence of cancer.^4^^,^^5^ In Sweden, campaigns to decrease tobacco smoking has been common since the 1970s and have resulted in one of the lowest rates of smokers in the European union.^6–8^

The background of this study was the intention to review the successful public prevention of tobacco smoking to decrease the risk of lung cancer. The hypothesis was that we should find decreasing incidence rate of lung cancer varying by sex and age, related to the changing smoking habits of the general population.

## Methods

Sweden has a cancer registry with mandatory reporting since 1958. We used incidence rates provided by the Swedish Cancer Register from 1970 to 2021. Such data are available online.^9^ We studied primary lung cancer (ICD7 1621) and stratified analyses according to histological cell type in squamous cell cancer (code 146), adenocarcinoma (code 096) and others. The latter mainly included cases coded as undifferentiated (code 196) and malignant epiteloid tumours (code 186). Code 186 started to be used by the register in the mid-1980s. We studied incidence rates in age-groups 40–84 years. The group 85+ constituted 7% of all lung cancer cases in 2021 and had a varying age distribution during 1970–2021. It includes a larger proportion of persons 95+ in recent years. The youngest ages were excluded as lung cancer is uncommon in ages below 40 years (0.6% in men and women in Sweden 2021). We estimated the total incidence rates per age group and calendar year by total number of cases in men and women divided by the corresponding population for the calendar year from data available from Statistics Sweden.^10^

Due to taxation on tobacco, there is information about the sale of tobacco products since 1856.^11^ The sold number of cigarettes per person above 15 years of age and year was available from 1916.^11–13^

The Public Health Agency of Sweden performs yearly surveys about the smoking habits in the Swedish population since 2004.^13^ Previously, data were surveyed sporadically by different organizations, but from mid-1970s more regularly. The surveys mostly registered smoking habits depending on age, calendar year and sex. Such data were retrieved from reports and web pages.

All data are based on open sources and there is no information linked to single individuals and no need for an ethical review.

## Results

### Smoking dose in the population and the occurrence of lung cancer

**Figure 1:**
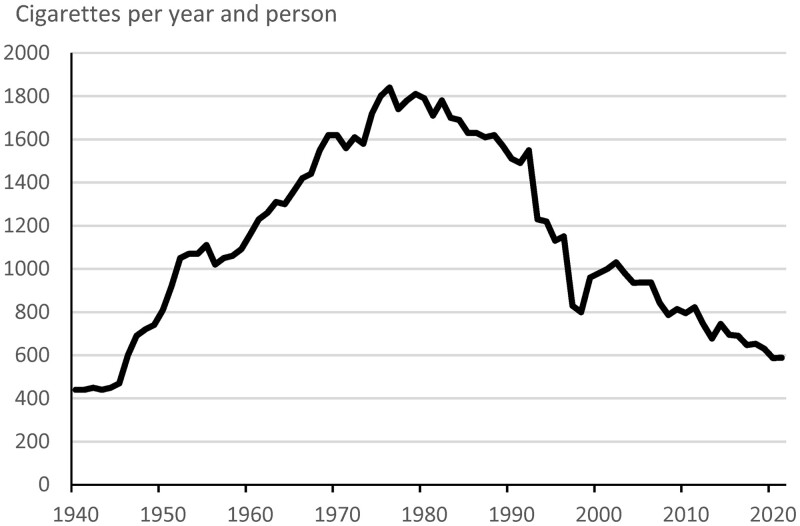
Average number of cigarettes sold per year and person over 15 years of age in Sweden between 1940 and 2021.

Among those above the age of 15 years, the sale of cigarettes per person was 280–440 cigarettes/year between the years 1916 and 1940. From year 1940, the sale increased from 440 cig/year until it peaked in 1976, at 1800 cig/year. Subsequently, the sale decreased to around 600 cig/year in 2021 (figure 1).^11–13^ There were in total 150 209 cases of lung cancer in persons aged 40–84 years in 1970–2021. There was a large increase among women, while the incidence in men was rather constant (Supplementary figure S1). The age distribution of persons with lung cancer changed, e.g. lung cancer cases 70–74 years old constituted 39% in 1970 and increased to 67% in 2021.

**Figure 2:**
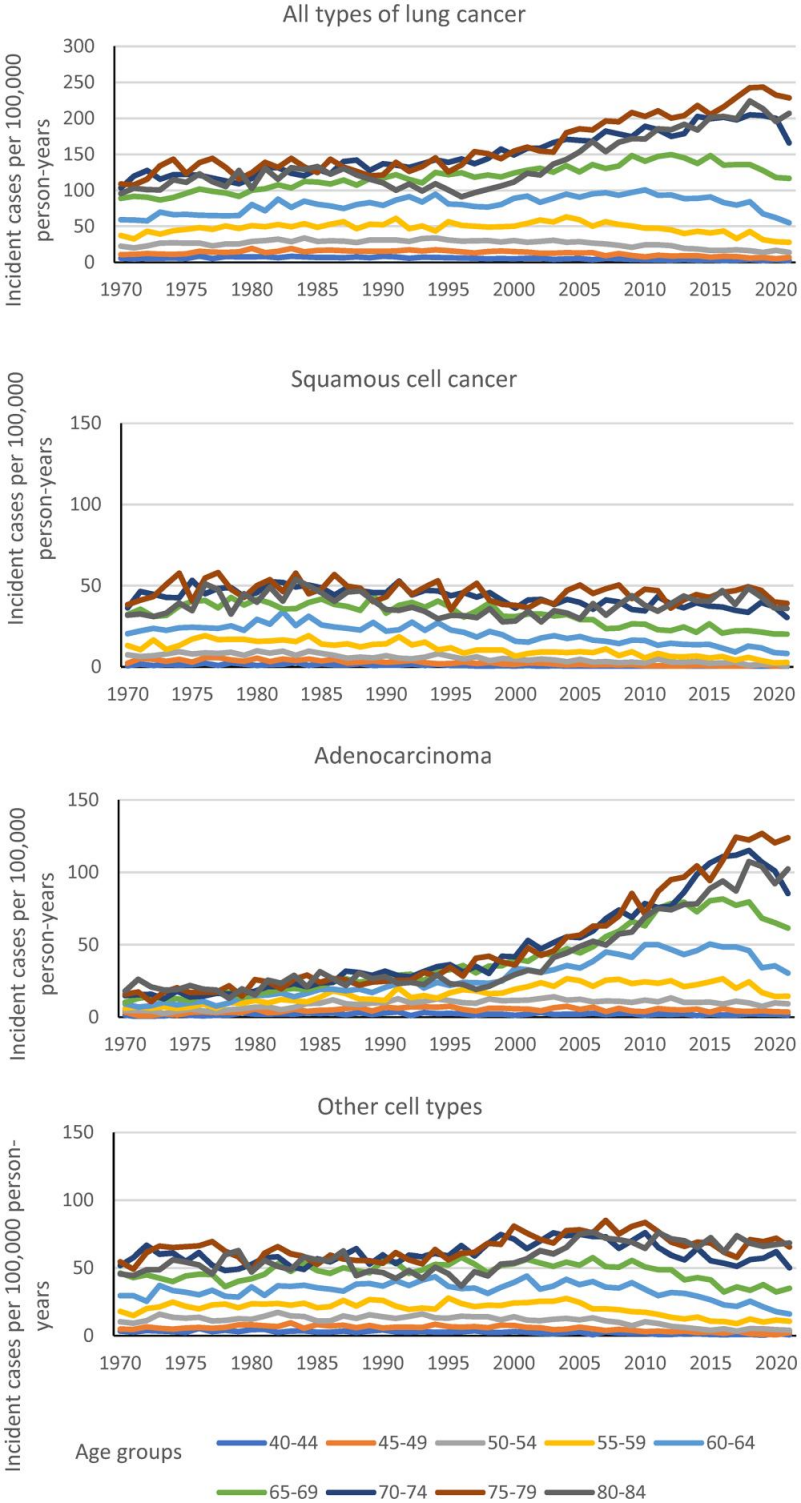
Incidence rates of all types of lung cancer, squamous cell cancer, adenocarcinoma and other cell types according to age 1970–2021 (cases per 100 000 persons and year).

The incidence rates for lung cancer (all types) decreased for the younger age groups from mid-1990s while it increased in the oldest age groups (figure 2). There was a decrease the last few years in age groups 55–74 years. The rates for squamous cell cancer were stable in the ages 75–84 years, while it decreased in younger age-groups from around 1980. The incidence for adenocarcinoma increased, especially in older ages. The trends for other cell types are more like that of squamous cell cancer, although there was an increase in mid-1990s in age groups 70–84 years.

### Sex, age, smoking habits and the occurrence of lung cancer

There have been large differences in smoking habits among men and women in Sweden. A survey in 1946 of 3000 Swedes above 18 years of age classified 83% of the women and 41% of the men as never-smokers.^11^ A similar survey in 1955 indicated that about 70–80% of the men were smokers (Supplementary table S1). In 1963 only 11% of women aged 50–69 years were daily smokers, compared to 46% among men (Supplementary table S2).^14^ The decline of smoking habits among men started in the 1970s, e.g. among men 55–70 years 40% were smokers in 1976 and 30% in 1983. The corresponding frequencies in women were 21 and 18%, respectively. Men and women aged 25–44 years had similar smoking habits in 1980s, while more women were smokers in aged 45–64 years from mid-1990s (Supplementary tables S3–S5 and figure S2b). Men and women of ages 65–84 years had similar smoking habits from around 2000, while the percentage of smokers were three times higher in men in the 1980s. The prevalence of daily smokers in Sweden between 1980 and 2000 is described in the supplement (Supplementary figure S2a–d).

The surveys from the Swedish Public Health Agency made it possible to also estimate the prevalence of ever smokers, while previous surveys focused on the current smoking habits, Table S4.^13^ Surveys during the 2004–2021 indicate that the prevalence of ever smokers were similar between men and women of all ages in 2021, while the prevalence was higher in men of age 65–84 years in 2004.

The smoking habits in different generations has been estimated from surveys in 1988/89 and 2004/05 and showed a similar decrease from a similar percentage in daily smokers of women and men born in the 1940s (from 34–35% to 21%).^15^ Among men born in the 1930s it decreased from 28% to 10% and decreased from 26% to 15% in women. It was estimated that 19% of women born in the 1920s were smokers in 1988/89 which decreased to 9% in 2004/05. The corresponding percentages for men were 25% and 8%, table S5.

**Figure 3:**
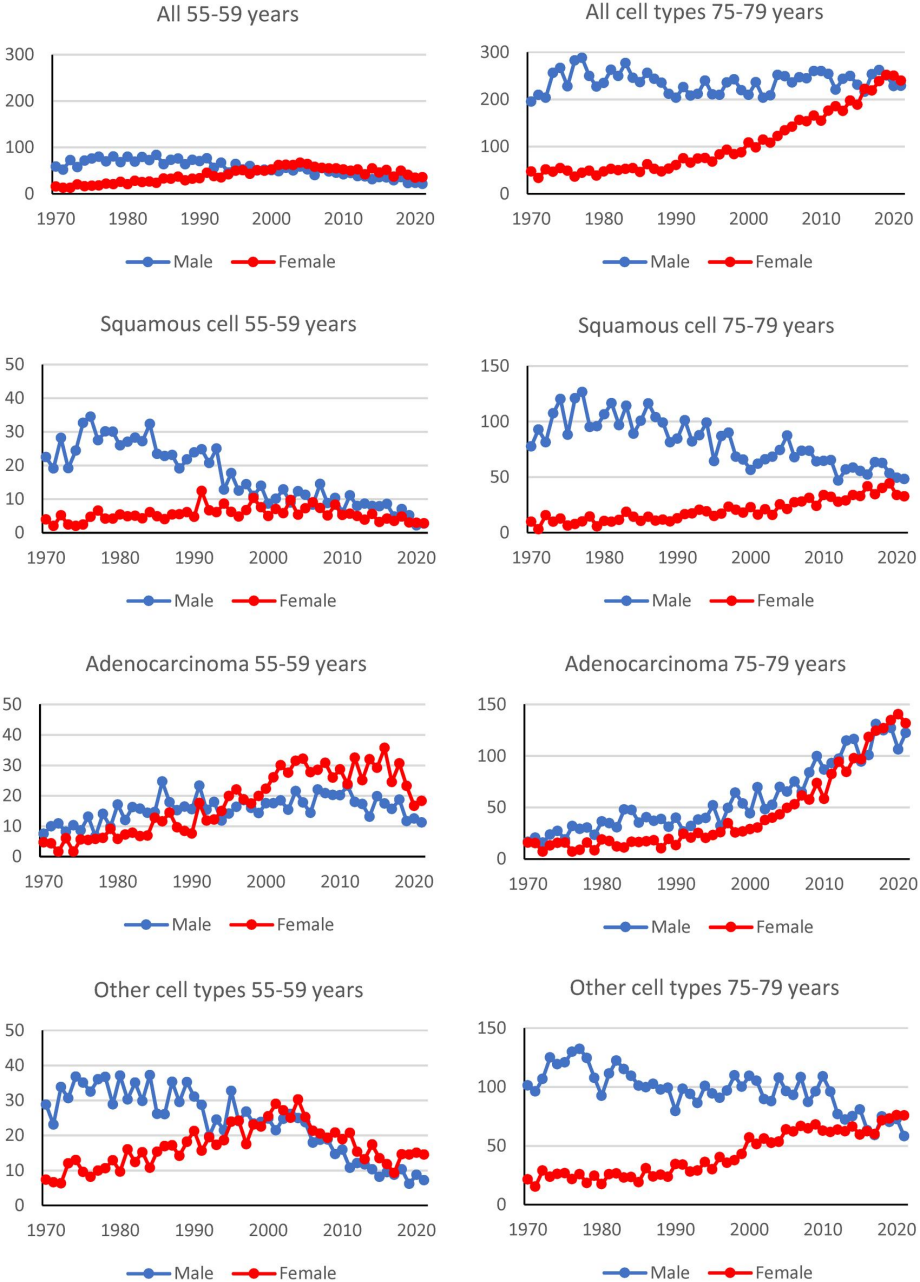
Incidence rates for lung cancer according to age, sex and time (cases/100 000 person-years). Notice the different scales on the vertical axes (incidence rates).

Over time lung cancer trends in men and women have converged towards similar lung cancer rates (all cell types, figure 3). The year of convergence for the 75- to 79-year-olds was around 2015, whereas for 55–59-year-olds convergence occurred earlier, around 1995. Incidence rates for ages 50–54, 60–64, 70–74 and 80–84 years are presented in the Supplementary figures S3–S6. However, there are stark differences between the cell types.

The incidence rates for squamous cell lung cancer within the age group of 55–59 years were higher in men compared to women until 1990s. Then, the incidence was similar in women and men figure 3). There was a decreasing trend in men and an increasing trend in women aged 75–79 years. The incidence rates were ten times higher for men in the 1970s. There were clear differences between men and women in the 1970s for all age groups (Supplementary figures S2–S5). For younger ages, the incidence rates were similar for men and women in the later period.

The incidence rates for adenocarcinoma were similar in women and men aged 75–79 years and increased between 1970 and 2021 (figure 3). For women aged 55–59 years the rates for adenocarcinoma were increasing until around 2010, while for men of this age there was an increase from 1970 to 1990. For ages 60–64 and 70–74 years the rates also increased 1970–2021 and were similar in men and women (Supplementary figures S2–S5).

In men aged 55–59 years there was a decreasing trend of the rates for other cell types from around 1990, while the rates for women of that age peaked around 2005. Men and women of age 75–79 years had similar rates from around 2015 (figure 3). The patterns in the other age groups were similar, i.e. a large difference between men and women in the early period and then a similar rate in later period (Supplementary figures S2–S5). The time when the rates were similar differs between ages. It occurs later in higher ages (Supplementary figures S2–S5).

## Discussion

The rather rapid decrease of risk of lung cancer after smoking cessation described in earlier studies,^2^^,^^3^ is not in accordance with our findings of a constant or even increasing trend among men aged 70–84 years, particularly in more recent years when the smoking prevalence is much lower than in the 1970s and 1980s. A review in 2007 concluded that the reduction of risks after cessation was similar regardless of type of lung cancer.^16^ However, a large proportion of Swedish men and women with lung cancer in older ages today have adenocarcinomas, which were rare in older men in the early 1970s compared to other cell types.

The benefits of smoking cessation in the Swedish population have had a lower impact than expected on the total occurrence of lung cancer due to the increasing occurrence of adenocarcinoma. For instance, if men and women aged 40–84 years had the same incidence rates today as in 1970 there would have been 2250.4 cases among men instead of 1695, and 544.4 cases among women instead of 2181.

### Methodological aspects

Changes in diagnostic procedures or reporting praxis are possible causes of bias. The Swedish Cancer Registry was established in 1958 and reporting is mandatory. Lung cancer is a serious disease, mostly diagnosed and treated in hospitals. In Sweden, treatments and care are almost free of charge and available both in public and private care. Thus, a major reporting bias is improbable.

The population is dynamic, i.e. some persons emigrate and there is immigration. The percentage of the population that is not born in Sweden has increased. It was 8% in women and 7% in men in 1982, 12% in women and 11% in men in 2000 and 20% in both men and women in 2021.^17^ It is also depending on age, e.g. it was 5% in 2000 in men aged 80–84 years and 11% in 2021. Thus, if immigrants have different smoking habits than Swedes, the tobacco consumption may be over- or underestimated and mostly influence findings in younger ages and late in the study period.

We have used two sources to estimate smoking habits; the sale of cigarettes taken from taxation data and smoking habits reported in interviews/questionnaires. The information of sale of tobacco as a measure of tobacco smoking may be biased if tobacco is smuggled or bought abroad. Statistics based on interviews during 2003–2017 indicate that 1% and 2% of the population has bought tobacco abroad and each person about 200 cigarettes.^16^ It has been estimated that around 10% of the consumption in 2012–2017 was from cigarettes bought abroad or smuggled.^17^

It may be difficult to get a representative sample of the population in surveys. The survey 2004–2021 by the Swedish Public Health Agency has a decreasing participation. The participation rate was 61% in 2004 among around 20 000 surveyed persons, while the rate was 44% in 2021 among around 40 000 persons.^13^ The postal survey in 1963 had a participation rate of 93%.^14^ A Swedish study of non-responders in questionnaire surveys indicates a higher prevalence of smokers among non-responders.^18^ Furthermore, persons with low socioeconomic status were more often non-responders. This implies that the smoking prevalence may be underestimated. However, men and women are measured with the same instruments, and we are not aware of any data indicating large differences in reporting biases of smoking habits between men and women.

The sales of cigarettes in Sweden peaked in 1976 with a yearly consumption of around 1800 cigarettes per person above 15 years. The average percentage of daily smokers 18–70 years in 1978–1982 was 33% in men and 31% in women. Assuming similar smoking habits in older ages would indicate that the smokers on average smoked 15 cigarettes per day (=1800/(0.32 × 365)). The daily consumption during the same period was 13 cigarettes per day for women and 15 cigarettes per day according to the survey of persons aged 18–70 years,^9^ that is, similar to the estimate based on sale. Around 600 cigarettes per person above 15 years were sold around 2020. An average prevalence of 5–10% daily smokers would indicate that each person smoked 16–32 cigarettes per day. This is considerably higher than the consumption reported in surveys 2003–2020 of daily smokers 17–84 years old, which decreased from 9 to 6 cigarettes per day.^19^ Thus, the comparison between sale of cigarette and surveys indicates that the consumption per smoker may be under-reported in 2020 and/or that non-responders more often are smokers.

Smoking tobacco mostly starts in the adolescence while cessation of smoking occurs at different ages; some succeed with a lasting cessation while some will relapse. Nevertheless, the yearly sale of cigarettes per person has from 1980 to 2021 decreased from around 1800–600, i.e. a decrease of about 67% indicating a considerably lower population dose compared to the 1980s.

Thus, the low prevalence of smokers in the 2000s reported by surveys is probably an underestimation but can hardly explain the absence of a rapid decline in occurrence of lung cancer after smoking cessation in older age groups. A possible explanation is that the decrease in risk by cessation of smoking depends on age at cessation and time since cessation.^20^ Furthermore, some smokers stop and start again. Thus, even if the population dose decreased, then the distribution of smokers according to age and time influence the occurrence of lung cancer.

### Incidence rates according to cell types and relation to changing smoking habits

Earlier research has indicated that the growth rate of lung tumours strongly depends on histopathology.^21^ Based on findings from repeated measurements of tumour growths in patients the average latency time from one cell to a detectable tumour was estimated to 13.2 years for adenocarcinoma, 7.2 years for squamous cell cancers and 2.4 years for oat cell cancers. The uncertainty was rather large, e.g. from 2.6 to 67.7 years for adenocarcinoma. Oat cell cancer is in our classification mostly like ‘other cell types’. The rather rapid decreased incidence in other cell types and squamous cell types is in accordance with a rapid growth and modest latency time. Thus, the different occurrence in men and women and converging time trends for other cell types and squamous cell cancer seem to be related to the changing smoking patterns. The rates for other cell types seem to be more similar in men and women some years earlier than for squamous cell types which are in accordance with a shorter latency time for other cell types.

Previous studies have indicated an increasing trend in adenocarcinomas.^22–26^ Wynder et al discussed if the cause was new types of cigarettes or changing smoking patterns.^22^^,^^23^ Lewis et al found increasing trends in older ages, but higher rates in men than in women.^24^ The Swedish incidence rates of adenocarcinoma have increased in both absolute measures and as proportion of the lung cancers. For example, they constituted around 10% of the cases in men 65–79 years old in 1970–1974 while around 50% of all male cases at these ages in 2016–2020 were adenocarcinomas. The incidence rates were around 15–20 cases per 100 000 person-years in the beginning of the study period in men and women 75–79 years of age and increased to about 120-135 cases per 100 000 around 2020, i.e. a six-fold increase. Similar rates among men and women indicate the similar causes and the similar exposure according to the level and time of exposure.

A possible explanation for the increased incidence of adenocarcinoma could be a decrease in competing risks. For example, former smokers may bypass earlier hazards experienced by current smokers and live long enough to suffer from smoking-induced cancers with longer latency times.

Tobacco smoking has changed over time not just in percentage of smokers. Tobacco smoke contains thousands of chemicals of which several are genotoxic.^27^ It has been suggested that tar is an important cause of lung cancer from tobacco smoke. Tar occurs mostly in the particles which are partly absorbed in the filter of a cigarette, while nitrosamines in the gaseous part of the smoke are not so much reduced by filters.^25^^,^^26^ Smoking Swedish men in the 1950s smoked pipe more often than women; the prevalence in men aged 15–67 years decreased from 26% to 11% 1971–1983 while it was around 1–3% in women.^11^ Information about the use of filter cigarettes in Sweden is sparse. A survey in 1963 found that among cigarette smokers 11% of the men and 29% of the women used filter cigarettes.^14^ A website reports that 70% of cigarettes sold in 1970s had filter^28^ ( Supplementary note 1). The increased use of filter cigarettes meant that the exposure to tar decreased.^24^ The use of filter cigarettes was positively associated with the increasing trend of adenocarcinomas in USA and Japan while non-filter cigarettes were positively correlated to squamous cell cancer.^29^ Our findings are like previous studies showing an increasing use of filter cigarettes and increasing occurrence of adenocarcinoma.^25^ Case-reference studies have indicated that the association between smoking and adenocarcinoma is weaker in women than men.^30^ However, those studies include probably populations that has smoked both filter and non-filter cigarettes. Thus, the change to filter cigarettes may have contributed to the increasing incidence of adenocarcinoma in Sweden. ‘Other cell types’ can be regarded as lower differentiated cancer that originates from more differentiated cell types. However, there may also be other causes to the increasing trend of adenocarcinomas in men and women that are hard to detect in a smoking population. Analysis of lung cancer adenocarcinomas from English ever-smokers indicated that around 25% lacked the typical smoking-mediated mutagenesis signature.^31^

In Sweden, men have used snus (smokeless tobacco, placed under the upper lip) in a much higher proportion than women especially some decades ago.^11^ Thus, it can be excluded as important cause to adenocarcinomas. Smoking of cannabis is illegal in Sweden and statistics of use are difficult to interpret. Surveys in 2004–2007 indicated the similar use in men and women, around 2%.^32^ A study of male Swedish conscripts found an increased risk for lung cancer and especially for adenocarcinomas which was attributed to the use of cannabis.^33^ A study of urinary samples from persons using both tobacco and cannabis showed increased levels of combustion products such as pyrene and fluorene, but not tobacco specific nitrosamines.^34^

Occupational industrial exposures are not probable causes to the increasing incidence of adenocarcinomas as a much lower percentage of Swedish women worked in industries than men. E.g. the percentage of women in the construction industry has been below 10% and women has a much lower risk for asbestos-related cancer such as mesothelioma than men.^35^ The concentration of radon in dwellings increased when the ventilation decreased, and the houses were sealed to decrease the cost of heating in early 1970s. However, the highly radon exposed population is too small to explain the change in rates.^36^

Outdoor air pollution is classified as a cause of lung cancer by IARC, and a recent study of mice indicated that inhaled particles could cause lung cancer, particularly adenocarcinomas, through a promotive mechanism.^37^^,^^38^ However, measurements of particles in the general Swedish environment indicate decreasing levels during the last decades which makes particles in the general environment a less probable cause.^39^^,^^40^

### Weaknesses and strength

This is an ecological study with its inherent weaknesses. On the other hand, it includes persons rarely included in cohorts, e.g. persons with addictions and homelessness. It also evaluates the impact of the smoking cessation intervention on the general population. The low prevalence of smoking in Sweden is a success as it has decreased the incidence of lung cancer in younger ages, figure 2. It has also other positive effects as, i.e. decreasing the risk of cardiovascular and respiratory diseases. However, the effect on the occurrence of lung cancer in the oldest ages is less successful. It would be of interest to document whether similar trends in lung cancer are present in other countries.

## Conclusion

The risk of lung cancer in the Swedish population several years after the smoking has steadily decreased is less favourable than previous studies have indicated, although the reported low prevalence of daily smokers in the Swedish population may be an underestimation. There is a similar increase in the incidence of adenocarcinoma in both men and women. The increasing use of filter cigarettes may have contributed, but other causes should be considered.

## Supplementary Material

ckae050_Supplementary_Data

## Data Availability

Data on incidence and incidence rates are available from https://www.socialstyrelsen.se/statistik-och-data/statistik/statistikdatabasen/. Data on population is available from https://www.statistikdatabasen.scb.se/pxweb/sv/ssd/. Key points Although the smoking habits in the Swedish population have decreased, the incidence rates of adenocarcinoma in the lungs have increased in older age groups. Comparing surveys of the population and taxation of cigarettes indicates that tobacco smoking habits has been underestimated by surveys in recent years. Men and women have similar incidence rates of adenocarcinomas although they have had different smoking habits.
