# Supplementary material for: Changing smoking habits and the occurrence of lung cancer in Sweden—a population analysis
Source: Eur J Public Health. 2024 Mar 22;34(3):566–71. doi: 10.1093/eurpub/ckae050 (PMC11161152; doi:10.1093/eurpub/ckae050)
Supplement: ckae050_Supplementary_Data [file ckae050_supplementary_data.pdf]

Supplement

Table S1 Prevalence of smoking (%) according to age in 1955 (1). Sample 1,415 men and 1438 women.

Age	Smokers		Ex-smokers		Never-smokers	
	Women	Men	Women	Men	Women	Men
15-19	40	51	4	8	56	41
20-29	47	80	5	6	49	14
30-39	79	79	7	9	44	12
40-49	33	72	4	14	63	14
50-64	21	79	4	14	75	7
65+	15	76	4	17	81	7

Table S2 Prevalence of current smokers (%) according to age in 1963 (N=55,000) (1).

Age	All smokers		Cigarette smokers	
	Women	Men	Women	Men
18-24	36	51	36	44
25-34	32	54	32	43
35-49	25	53	25	36
50-69	11	46	11	26

Table S3 Current smokers 2004-2021 according to age and sex, (2)

	16-29 yr	16-29 yr	30-44 yr	30-44 yr	45-64 yr	45-64 yr	65-84 yr	65-84 yr
	Women	Men	Women	Men	Women	Men	Women	Men
**2004**	15.6	9.9	17.6	14.2	25.1	18.8	12.2	10.6
**2005**	14.8	9.2	16.5	11.0	22.4	17.1	11.1	9.8
**2006**	13.3	7.2	13.6	11.3	20.6	17.8	10.9	11.5
**2007**	14.0	8.6	13.5	9.3	22.2	17.6	13.3	10.8
**2008**	11.9	7.6	11.7	11.4	19.2	15.0	11.3	9.3
**2009**	10.3	9.9	11.5	8.4	18.4	16.3	10.8	9.8
**2010**	12.5	8.6	11.5	8.8	17.1	17.6	10.3	10.9
**2011**	12.7	7.7	8.5	9.1	16.6	13.2	10.5	10.0
**2012**	11.5	7.9	9.4	9.0	16.2	13.9	11.1	9.7
**2013**	12.2	7.1	7.4	9.3	13.7	14.6	11.4	10.4
**2014**	8.9	7	6.5	8.5	16.3	11.7	11.7	8.5
**2015**	10.9	5.8	7.5	7.0	13.8	11.7	11.8	8.4
**2016**	7.6	7.6	8.1	5.5	12.8	9.1	8.3	9.6
**2018**	5.4	4.6	4.7	5.6	9.9	9.3	8.4	7.6
**2020**	4.3	4.2	5.1	6.8	8.5	8.3	8.8	6.8
**2021**	3,5	2,6	3,7	6,6	8,6	7,3	7,7	6,5

Table S4 Prevalence (%) of daily smokers and ever smokers (2004 and 2021 (2) and 2000 and 1980 (3))

		**Daily smokers**					**Ever smokers**	
Age	Year	1980	2000	2004	2021		2004	2021
30-44	Women	40[a]	23[a]	18	4		41	20
	Men	43[a]	15[a]	14	4		40	20
45-64	Women	24	26	25	9		52	31
	Men	37	21	19	7		53	26
65-84	Women			12	8		33	40
	Men			11	7		51	44
65-74	Women	14	15					
	Men	32	16					
75-84	Women	4	9					
	Men	25	12					

a/ 25-44

Table S5 Daily smokers (%) according to birth cohort in 1988/1989 and 2005/2005 estimated from surveys (4).

**Birth cohort**	**Prevalence (%)**				
	**Women**			**Men**	
	**1988/89**	**2004/5**		**1988/89**	**2004/5**
1900-09	5			22	
1910-19	10			22	
1920-29	19	9		25	8
1930-39	26	15		28	10
1940-49	35	21		34	21
1950-59	34	24		30	21
1960-69	34	19		22	13
1970-79		16			12
1980-89		16			12

Table S6. Cases of lung cancer in 2021 stratified according to age and sex and a counterfactual analysis estimating the number of cases in 2021 assuming the same incidence rates as in 1970.

Age	Women		Men	
	2021	Counter-factual	2021	Counter-factual
40-44	10	13.2	5	23.1
45-49	27	28.0	18	41.2
50-54	54	34.7	38	118.6
55-59	115	51.5	70	193.9
60-64	189	54.7	124	292.0
65-69	365	73.8	266	426.7
70-74	522	106.4	368	490.1
75-79	587	115.8	516	441.0
80-84	312	66.3	290	223.8
**Total**	**2181**	**544.4**	**1695**	**2250.4**

Figure S1. Cases of lung cancer per year in persons 40-84 years 1970-2021.

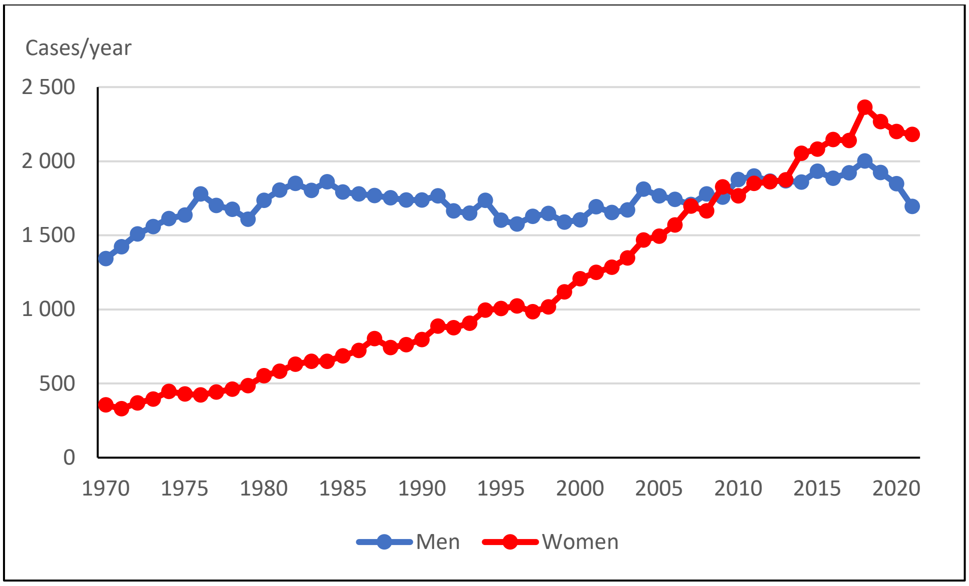

Figure S2 a-d. Daily smokers (%) according to age (3).

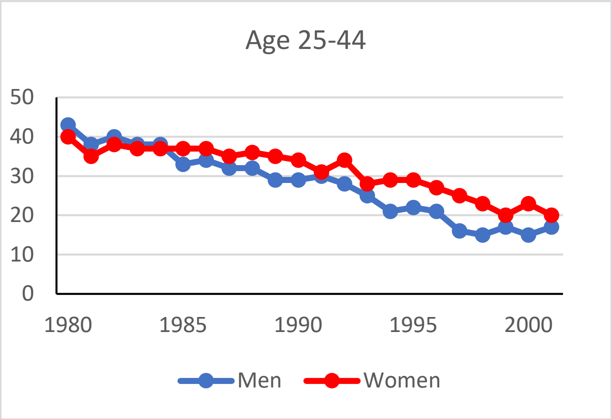

(a)

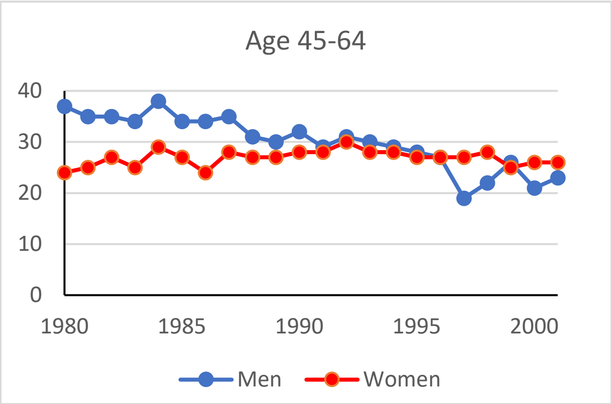

(b)

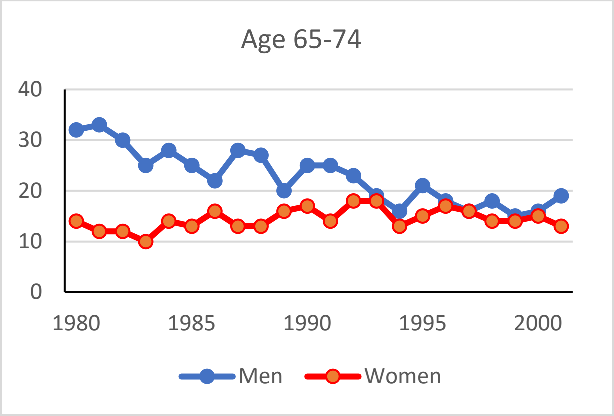

(c)

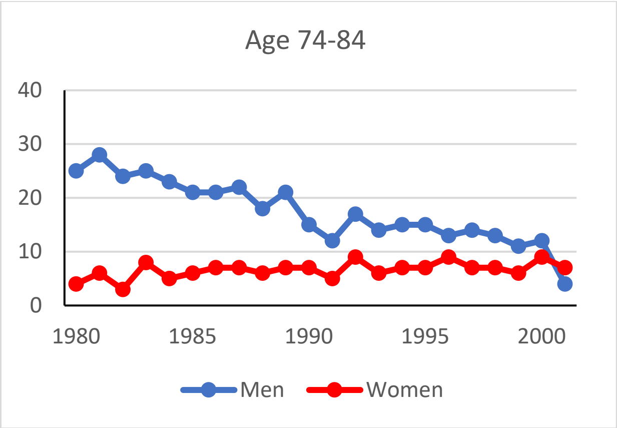

(d)

Figure S3a Incidence rates for men and women 50-54 years of age, all cell types.

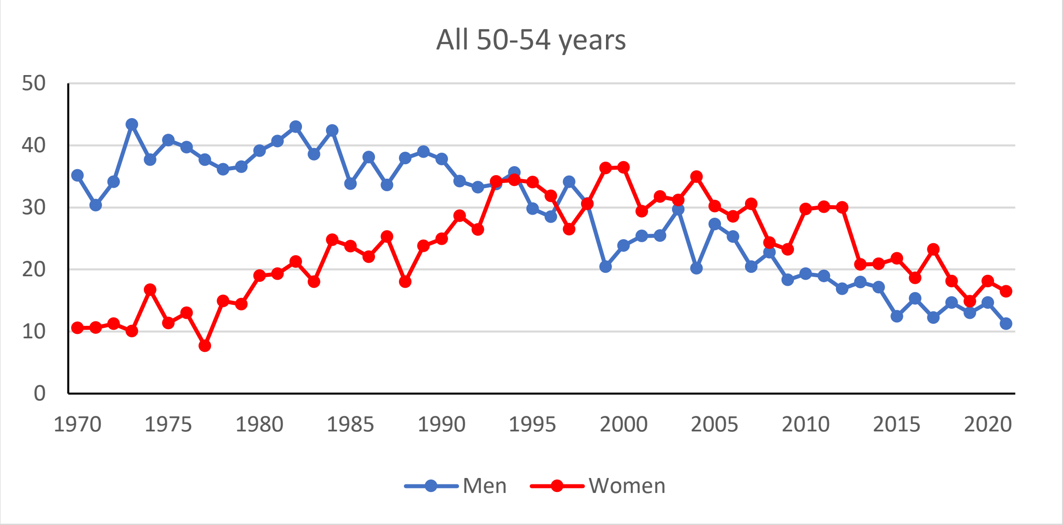

Figure 3 b-d Incidence rates for men and women 50-54 years of age according to cell types.

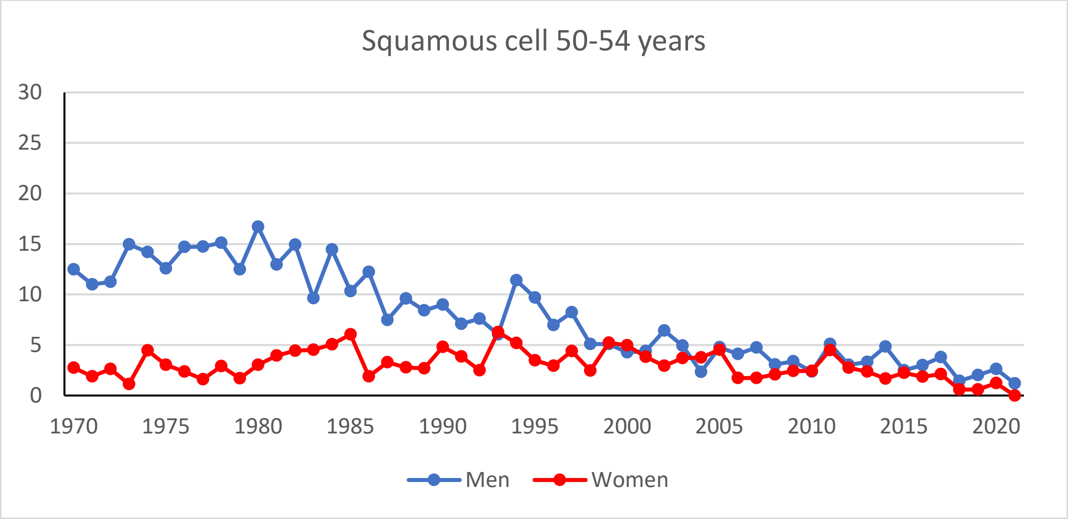

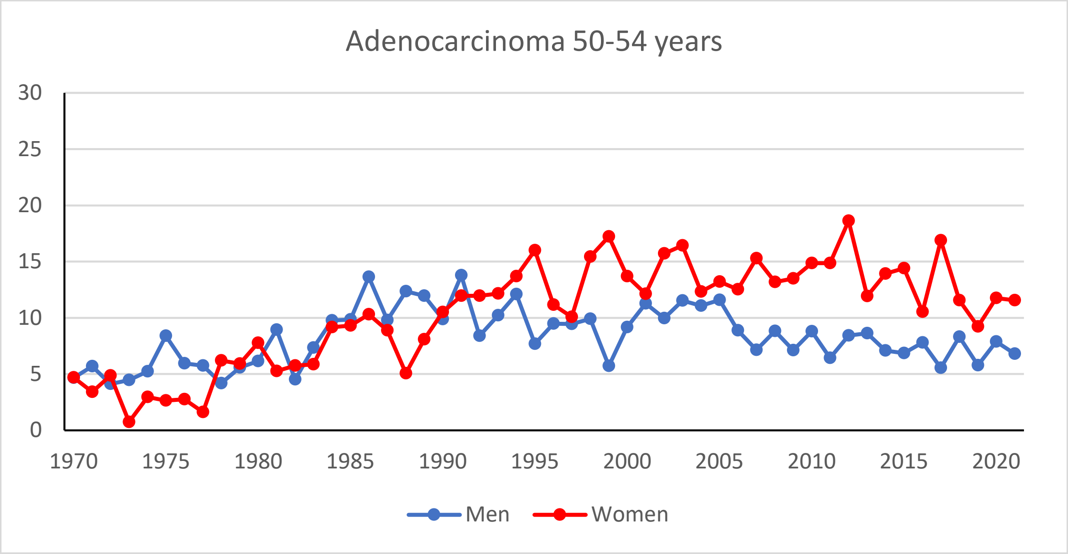

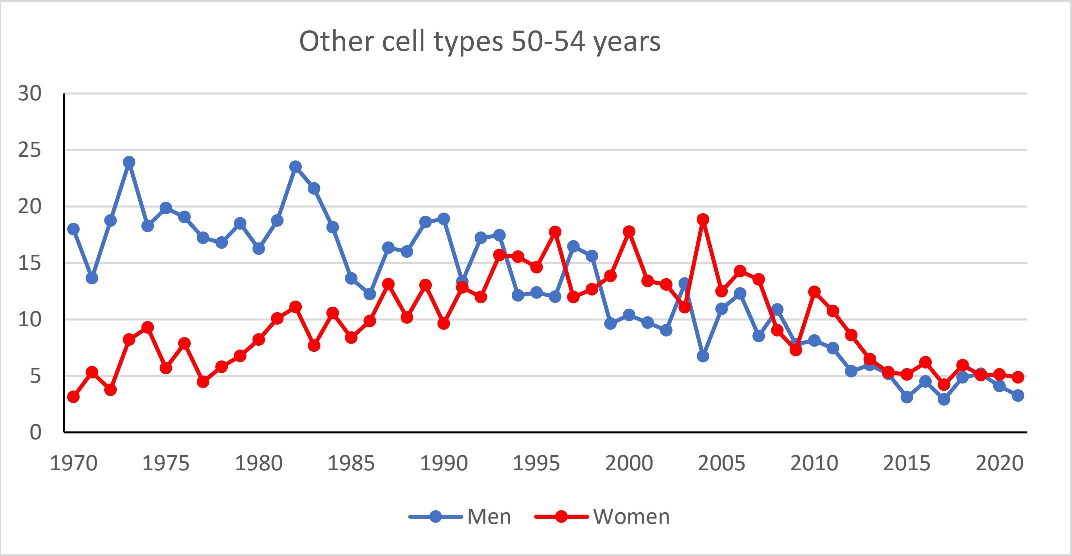

Figure S4a, Incidence rates for men and women 60-64 years of age, all cell types.

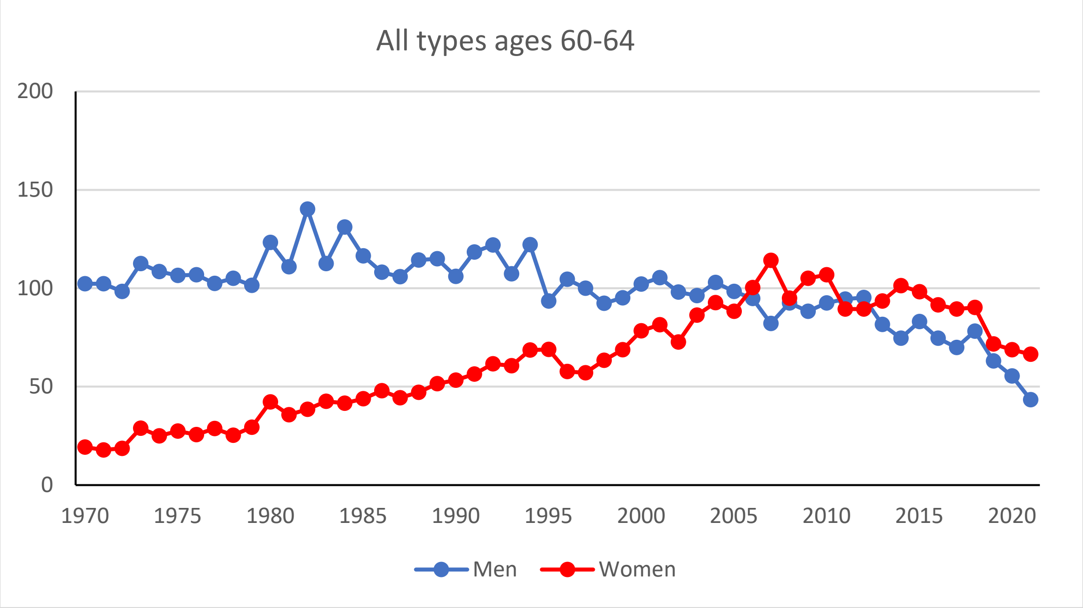

Figure S4b-d, Incidence rates for men and women 60-64 years of age according to cell types.

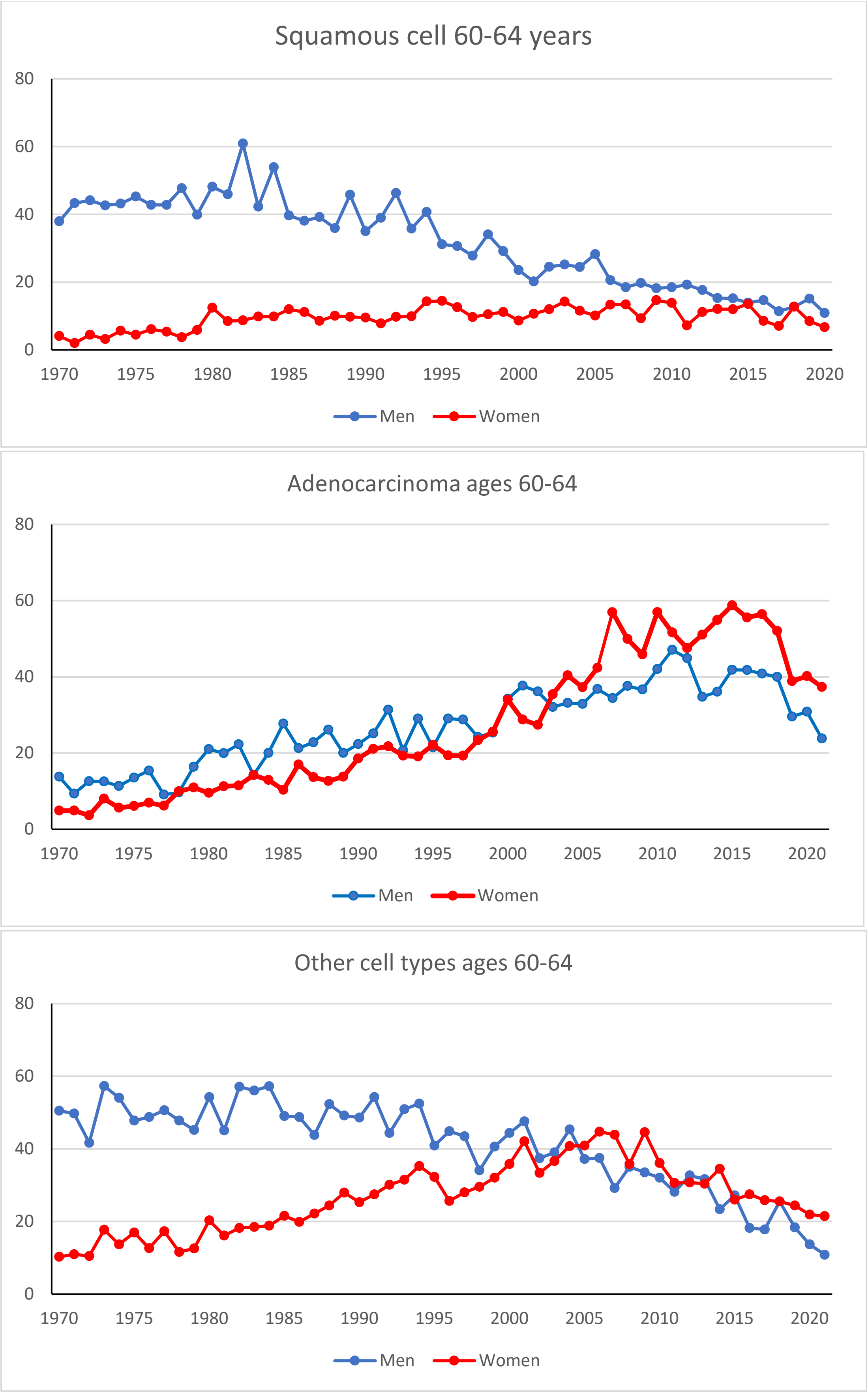

Figure S5a, Incidence rates for men and women 70-74 years of age, all cell types.

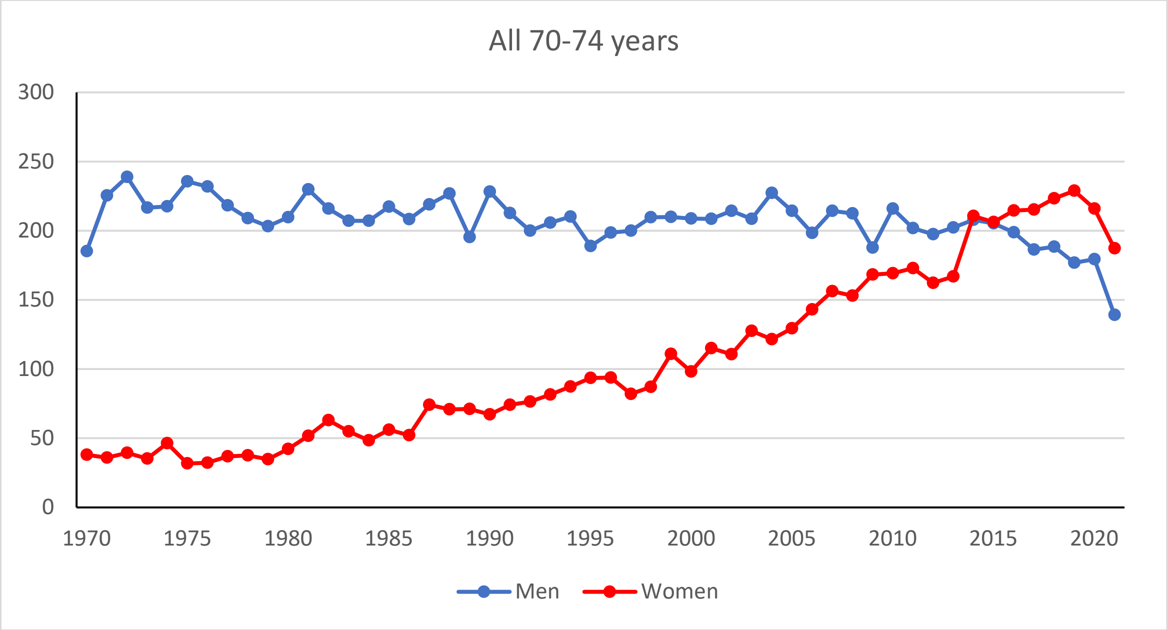

Figure S5b-d, Incidence rates for men and women 70-74 years of age according to cell types.

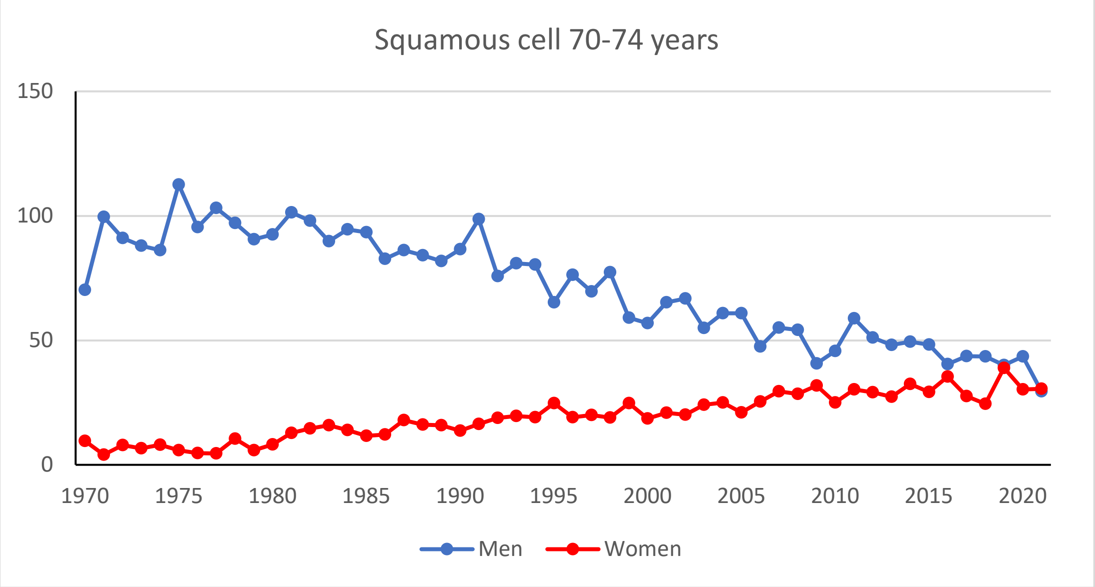

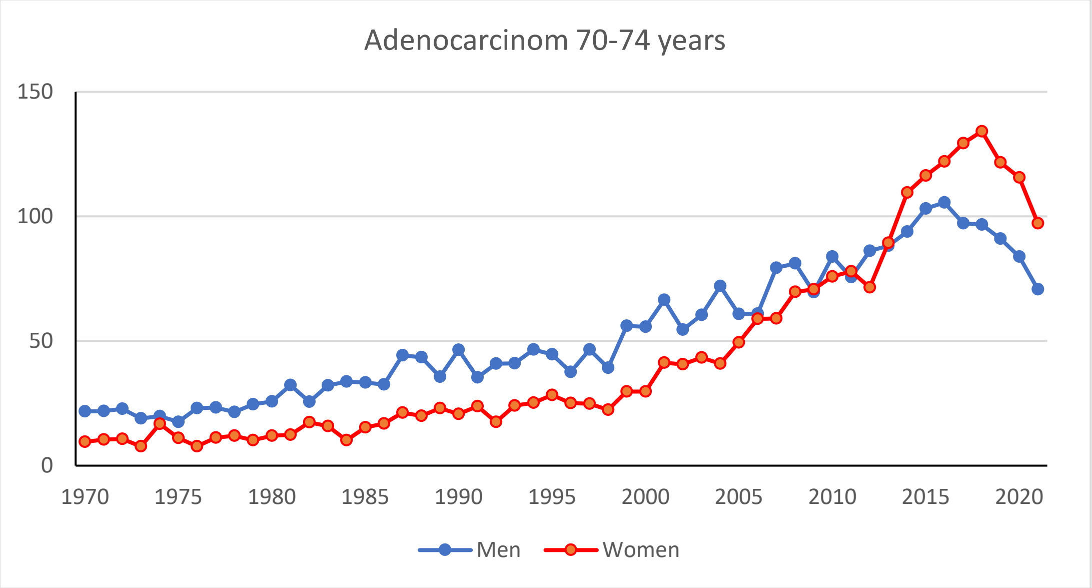

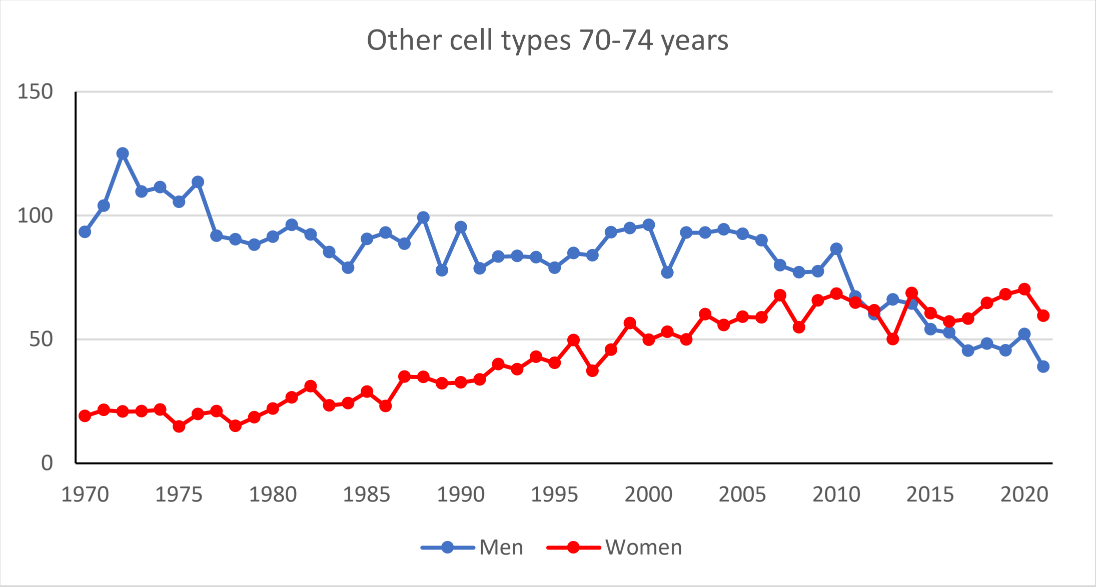

Figure S6a, Incidence rates for men and women 80-84 years of age, all cell types.

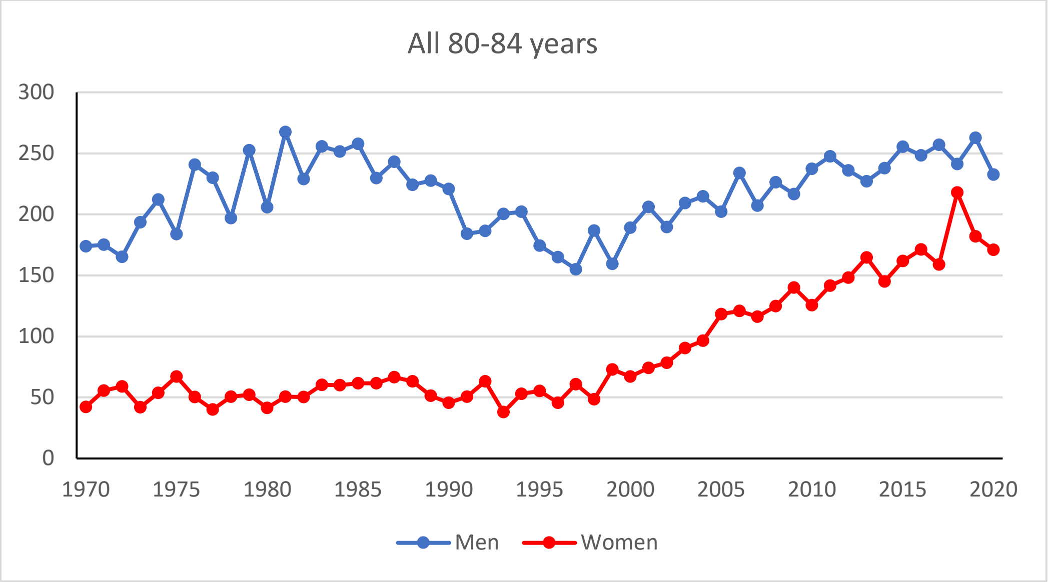

Figure S5b-d, Incidence rates for men and women 80-84 years of age according to cell types.

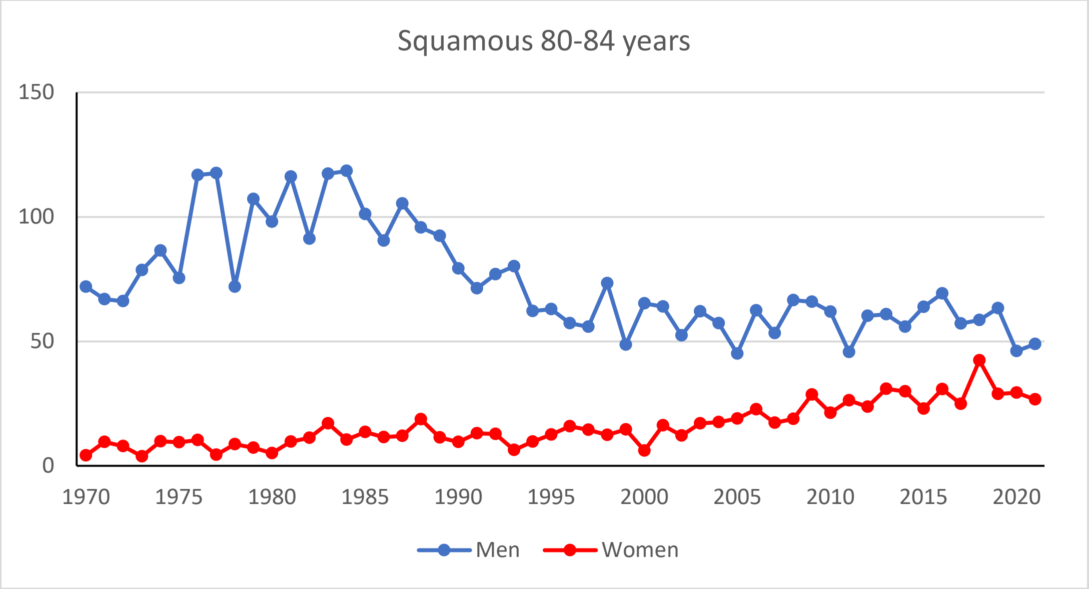

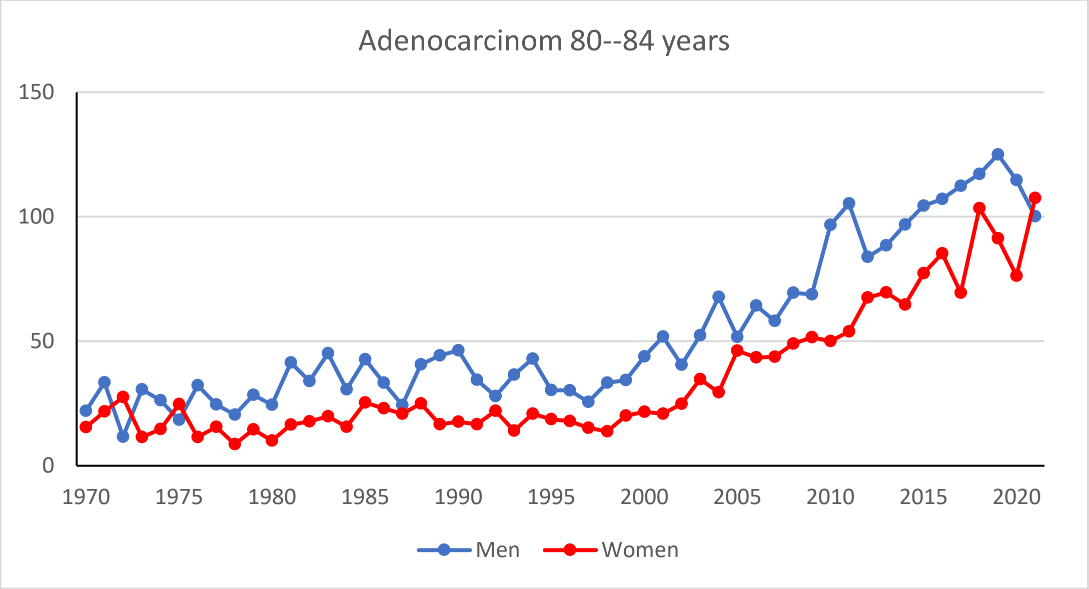

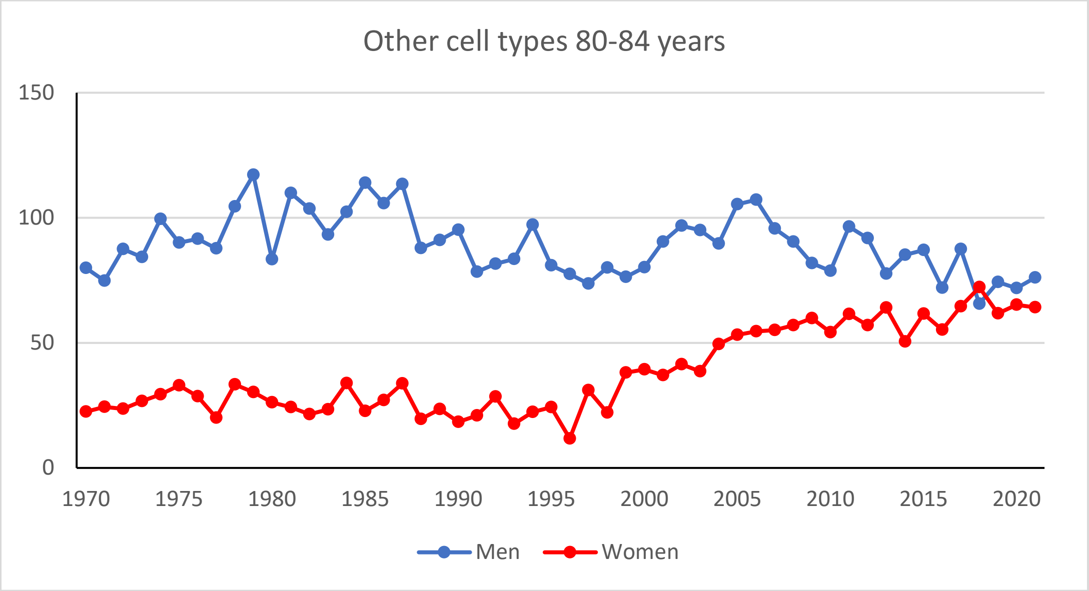

<u>Note 1</u>:

It has been difficult to find accurate information about the use of filter cigarettes in Sweden and the sources of the percentages on the website is not publicly available. Filter cigarettes can change the risk of lung cancer in several ways. It decreases the amount of tar but increase some other substances. The filter may also contain substances of importance for the risk of cancer. The filter also changes the size of the particles in the inhaled smoke to smaller sizes. That influence where the particles are deposited, smaller sizes means that the particles are deposited in distant parts of the lung. Squamous cell lung cancer is more often situated in larger bronchi, while adenocarcinomas are more often situated in distant parts of the lung.

References

1. Tobaksvanor i Sverige. En översikt och analys. (Tobacco habits in Sweden. An overview and analysis.) [Socialstyrelsen redovisar 1986:9]. Stockholm: Socialstyrelsen,1986.
2. Website: Folkhälsomyndigheten (Public Health Agency of Sweden): https://www.folkhalsomyndigheten.se/folkhalsorapportering-statistik/statistik-a-o/ovrig-statistik-a-o/tobaksbruk/ [Accessed 2022/07/05].
3. Drogutvecklingen i Sverige 2003. (Use of drugs in Sweden 2003). Centralförbundet för alkohol- och narkotikaupplysning. Stockholm; 2003. Report No 71.
4. Danielsson M, Gilliam H, Hemström Ö. Tobaksvanor och tobaksrelaterade besvär. (Tobacco habits and tobacco-related problems.) In: Danielsson M, editor. Folkhälsorapport 2009. Stockholm: Socialstyrelsen; 2009. p. 291-310.
